# Perceptions and satisfaction of a mandatory continuing professional development programme amongst Aotearoa New Zealand podiatrists

**DOI:** 10.1186/s13047-021-00492-6

**Published:** 2021-09-08

**Authors:** Matthew Carroll, Angela Brenton-Rule, Hannah Jepson, Prue Molyneux

**Affiliations:** 1 Department of Podiatry, School of Clinical Sciences, Faculty of Health & Environmental Sciences, Auckland University of Technology, Private Bag 92006, 1142 Auckland, New Zealand; 2 Active Living and Rehabilitation: Aotearoa New Zealand, Health and Rehabilitation Research Institute, School of Clinical Sciences, Auckland University of Technology, Auckland, New Zealand

**Keywords:** Podiatry, Continuing professional development, New Zealand

## Abstract

**Background:**

Aotearoa New Zealand (NZ) registered podiatrists are required to participate in a mandatory continuing professional development (CPD) programme. This study investigated podiatrist’s perceptions and satisfaction surrounding mandatory CPD requirements following the implementation of a new 2-year CPD programme.

**Methods:**

A cross-sectional study of NZ registered podiatrists was conducted between October 9th and December 9th, 2020. Data was collected using a web-based survey. The 39-item survey included questions to elicit participant characteristics, perceptions of CPD, difficulties undertaking CPD, and satisfaction with the new CPD programme. The survey findings were reported using descriptive statistics and conventional content analysis.

**Results:**

One hundred and thirty-four podiatrists completed the survey. Most respondents worked in private practice (*n* = 107, 80 %), were in full-time employment (*n* = 83, 62 %), and had greater than 16 years of work experience (*n* = 73, 54 %). Respondents agreed it was important to engage in CPD (*n* = 126, 94 %) and reported that knowledge gained from CPD contributed to their daily work (*n* = 78, 58 %). 44 % (*n* = 58) reported difficulties keeping up to date with CPD. The main barriers to CPD participation reported were workload (*n* = 90, 67 %) and lack of time (*n* = 84, 63 %). Three categories (understanding the CPD programme; access to CPD; and time to complete CPD) were identified from the qualitative analysis to describe why it was difficult to meet CPD requirements.

**Conclusions:**

NZ podiatrists value CPD and are satisfied with most aspects of the mandatory CPD programme apart from the hours attributed to compulsory activities. The current approach to cultural safety CPD requires revision, with a move away from a time-based approach to a system that promotes an understanding and relevance to practice. Lack of time, practice workload, financial barriers, geographical location, and employment context were factors that influenced a practitioner’s ability to engage in CPD. Facilitation of CPD activities that are flexible to ensure relevance to the practitioner’s specific work within their scope of practice, and that can occur in the workplace environment, may address barriers and increase engagement with to CPD activities.

**Supplementary Information:**

The online version contains supplementary material available at 10.1186/s13047-021-00492-6.

## Background

To ensure safe practice, high quality of care, and continuing professional improvement, the contemporary podiatry practitioner requires a unique combination of knowledge, clinical expertise, technical abilities, and communication skills. Developing and maintaining these skills is challenging in a rapidly evolving practice environment with continual technological advancements, heightened patient expectations, and increasing accountability. Continuing professional development (CPD) plays a role for practitioners in maintaining and adapting their knowledge and skills to support a culture of lifelong learning.

In Aotearoa New Zealand (NZ), mandatory CPD was introduced for podiatrists in 2004 with the introduction of the Health Practitioners Competence Assurance Act 2003 (The Act). The Act provides a framework for the regulation of NZ healthcare providers [1]. The principal purpose of The Act is: “to protect the health and safety of members of the public by providing mechanisms to ensure that health practitioners are competent and fit to practise their professions.” Under law, Government appointed responsible authorities have a role in regulating health professions to ensure The Act is upheld. The Podiatrists Board of New Zealand (PBNZ) is the responsible authority for podiatry. Among other responsibilities set out in Sec. 118 of The Act, the PBNZ must recognise, accredit, and set programmes to ensure the ongoing competence of registered podiatrists. To meet this obligation, the PBNZ has designed a recertification framework which requires all NZ podiatrists who hold an annual practising certificate (APC), to actively engage in CPD. This CPD framework is known as the PBNZ CPD recertification programme (henceforth referred to as the CPD programme). This CPD programme is relevant to all NZ podiatrists registered within the scope of “podiatrist”. NZ podiatrists who practice within the scope of “Podiatric Surgeon” have CPD requirements additional to the CPD programme.

In January 2018 the PBNZ implemented a new 2-year cyclical CPD programme [2]. The first cycle ran between January 1st 2018 and December 31st 2020. The new CPD programme was a substantial departure from the previous 4-year CPD framework that was in place from 2004 to 2017. Significantly, the new CPD programme moved to an online platform allowing all documents to be electronically uploaded and attainment of hours to be automatically recorded. The 2-year CPD programme consists of four activity categories (1) compulsory activities (infection control, wound management, cultural safety), (2) professional communication activities, (3) professional learning activities, and (4) basic life support (see Additional File 1 or https://podiatristsboard.org.nz/practitioners/cpd-requirements/ for full details). Within each category, the practitioner must achieve a minimum number of hours related to a broad base of activities. The CPD programme requires NZ podiatrists to engage in a minimum of 40 h CPD per 2-year cycle.

Compared to CPD requirements in the United Kingdom (UK) and Australia, NZ is more prescriptive as to the hours and categories of CPD that practitioners are expected to undertake. Although the NZ and Australian CPD programmes both require a minimum of 40 h per 2-year CPD cycle, NZ is alone in having minimum hour requirements in compulsory activities (wound care, infection control, cultural safety). This is in direct contrast the UK CPD programme, which does not set the number of hours that practitioners must complete to achieve their CPD requirements. However, like Australia and the UK, the CPD completed is expected to focus on aspects of podiatry practice that is relevant to the practitioner and aims to improve podiatric service delivery.

As engagement in CPD is mandatory for all NZ podiatrists who hold an APC, it is important to know whether it is achieving its objectives and gain a clear understanding of podiatrists’ perceptions of CPD and satisfaction with the current CPD programme. Therefore, the first objective of this study was to investigate the perceptions of NZ podiatrists towards CPD. The second objective was to investigate how satisfied NZ podiatrists were with the new CPD programme. Determining the level of satisfaction with the new CPD programme is important firstly, to see if the CPD programme is valued within the profession and secondly, to enable quality improvements to be made to the CPD programme.

## Methods

This cross-sectional observational study utilised a web-based survey. The anonymous electronic survey was implemented between October 9th and December 9th, 2020 using the Qualtrics XM, Provo, UT, software package. An email invitation to participate was sent to all registered podiatrists by the Registrar of the PBNZ. The email contained a URL link enabling access to the online survey. On clicking the URL link, respondents were directed to the participant information sheet detailing the purpose of the study, the length of the survey, how data would be stored, details of how anonymity was ensured, and contact details of the investigators. Consent for participation was implicit with submission of the survey. Anonymous responses were enabled in the Qualtrics security settings, ensuring respondents’ IP addresses, location data, and contact information were not recorded. No incentives were offered to aid survey participation. Study protocols were approved by the Auckland University of Technology Ethics Committee (AUTEC 20/280).

A draft survey was piloted with 10 NZ registered podiatrists. The pilot group respondents were from diverse clinical backgrounds (high-risk foot management, musculoskeletal practice, general practice, and academia). The draft survey was sent to the pilot group through an anonymous survey link via the Qualtrics platform. All pilot group members completed the online survey and provided written feedback on the survey questions. Based upon feedback, questions and wording within the online survey were amended to produce the final survey.

The final survey contained 39 items. Questions 1–7 related to participant characteristics including years practicing, age, employment status, and geographical area of practice. Other items included general perceptions of CPD (questions 8–12), satisfaction with the CPD programme, time spent on CPD, and difficulties undertaking the CPD programme (questions 13–16). The remaining items related to satisfaction with each of the four CPD categories: compulsory activities (questions 17–21); professional communication activities (questions 22–28); professional learning activities (questions 29–34); and basic life support (questions 35–39). Question back-tracking was enabled to allow respondents to review and change their answers, however, respondents were unable to make multiple submissions. No timeframe was used as a cut-off point for survey completion. All survey data was reported in accordance with the Checklist for Reporting Results of Internet E-Surveys (CHERRIES) [3] (Additional File 2).

### Data analysis

Participant responses were included in the final analysis if all survey questions were completed. Open-ended responses were categorised for the purposes of data analysis. All categorical data was described as number (n) and percent (%). Likert scale data categories were combined for final analysis. ‘Agree’ and ‘strongly agree’ responses were recorded as ‘agree’; ‘strongly disagree’ and ‘disagree’ responses were recorded as ‘disagree’; ‘extremely satisfied’ and ‘satisfied’ responses were recorded as satisfied; and ‘extremely dissatisfied’ and ‘dissatisfied’ responses were recorded as ‘dissatisfied’. Open-ended responses relating to difficulties completing CPD requirements (question 16) were consecutively compiled in an unstructured transcript document. A conventional content analysis approach was adopted to analyse the transcript data. This approach aims to describe a phenomenon, in this case experiences with a new CPD system, where research on the area is limited. The goal of content analysis is to classify a large amount of text into categories that represent similar meaning [4]. In conventional content analysis the researcher avoids using preconceived categories, instead allowing new insights to be developed. Data coding was inductive or data-led, meaning that the data itself was the starting point for analysis. The qualitative analysis was conducted by one researcher (ABR) and corroborated by (MC).

## Results

### Participant characteristics

In total 165 survey responses were received within the 2-month study period. This represented a 36 % response rate with 454 NZ podiatrists holding an annual practising certificate at the date of the survey closing. Thirty-one surveys were incomplete, and therefore excluded, leaving a sample of 134 full responses for final analysis. Most respondents worked in private practice (*n* = 107, 80 %), were in full-time employment (*n* = 83, 62 %), ang had greater than 16 years of work experience (*n* = 73, 54 %). Table 1 details the participant characteristics related to: time at workplace, work duration, place of work, work location, highest level qualification, and intention to start a formal qualification in the next 5 years.

**Table 1:** Participant characteristics

**Time at workplace, n (%)**	
Full time	83 (62)
Part-time (< 18.5 h per week)	11 (8)
Part-time (> 18.5 h per week, but less than 40)	40 (30)
**Work duration, n (%)**	
0–2 years	6 (4)
3–5 years	14 (10)
6–10 years	24 (18)
11–15 years	17 (13)
16–20 years	12 (9)
> 21 years	61 (46)
**Place of work, n (%)**	
Private practice	107 (80)
District Health Board	11 (8)
Education	6 (11)
Private practice, District Health Board	5 (4)
Administration	2 (1)
Education, Research	1 (1)
Industry/commercial	1 (1)
Private practice, Education	1 (1)
**Work location, n (%)**	
Auckland	45 (33)
Canterbury	25 (19)
Wellington	16 (12)
Waikato	13 (10)
Otago	7 (5)
Bay of Plenty	6 (4)
Northland	6 (4)
Manawatū-Whanganui	5 (4)
Taranaki	4 (3)
Southland	2 (1)
Hawkes Bay	2 (1)
Gisborne	1 (1)
Marlborough	1 (1)
Nelson	1 (1)
**Highest level qualification, n (%)**	
Bachelors degree	62 (46)
Diploma	23 (17)
Bachelors with Honours	17 (13)
Postgraduate diploma	15 (11)
Masters degree	6 (4)
Postgraduate certificate	6 (4)
Doctoral degree	5 (4)
**Intention to start formal qualification in the next 5 years, n (%)**	
Maybe	40 (30)
No	69 (51)
Yes	15 (11)
Currently undertaking a further qualification	10 (7)

### Perceptions and satisfaction with CPD

#### General CPD

**Fig. 1:**
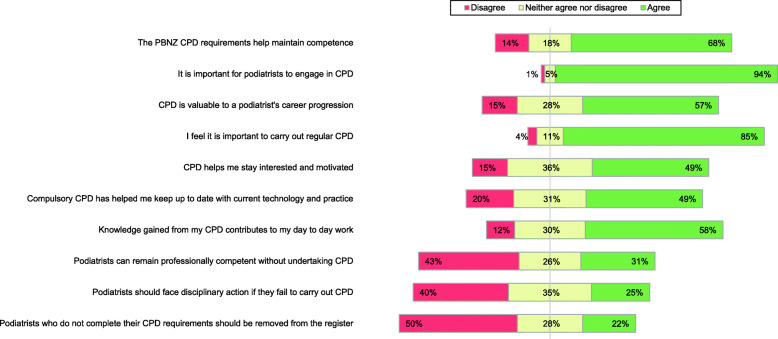
General perceptions of CPD

Figure 1 details general perceptions of CPD. Nearly all respondents agreed that it is important to engage in CPD (*n* = 126, 94 %). The majority agreed that the CPD programme requirements help maintain professional competence (*n* = 91, 68 %), and that it is important to carry out regular CPD (*n* = 114, 85 %). Most respondents agreed that CPD is valuable to career progression (*n* = 77, 57 %) and contributes to their daily work (*n* = 78, 58 %). Many agreed that CPD helps them to stay interested and motivated (*n* = 66, 49 %), and keeps them up to date with technology and practice (*n* = 66, 49 %). When asked if podiatrists could remain professionally competent without undertaking CPD, there was no dominant response with 43 % (*n* = 57) disagreeing and 31 % (*n* = 42) agreeing. One quarter of respondents (*n* = 33, 25 %) agreed that podiatrists should face disciplinary action for non-compliance with CPD requirements and 22 % (*n* = 32) agreed that podiatrists should be removed from the register for failing to comply with the CPD programme.

#### PBNZ CPD programme

**Fig. 2:**
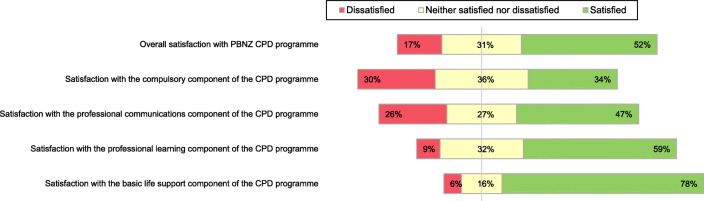
Satisfaction with the PBNZ CPD programme

Figure 2 details respondent’s perceptions of, and satisfaction with, the PBNZ CPD programme. Most respondents indicated they did not find it difficult to meet the requirements of the mandatory CPD programme (*n* = 94, 70 %), with most spending an average of 1-4 h per month towards their CPD activities (*n* = 63, 84 %). 52 % of respondents were satisfied with the PBNZ CPD programme (*n* = 70). The compulsory component of the CPD programme drew the most dissatisfaction (*n* = 40, 30 %). Approximately half of respondents were satisfied with the professional communications component (*n* = 63, 47 %), most were satisfied with the professional learning component (*n* = 79, 59 %), and the majority were satisfied with the basic life support component (*n* = 104, 78 %). Internet-based learning was the preferred format, with 31 % (*n* = 111) of respondents reporting they were comfortable with accessing online CPD resources. The majority reported that online CPD saved them time (*n* = 108, 81 %) and allowed them to plan CPD more efficiently (*n* = 81, 60 %).

#### Time requirements

**Fig. 3:**
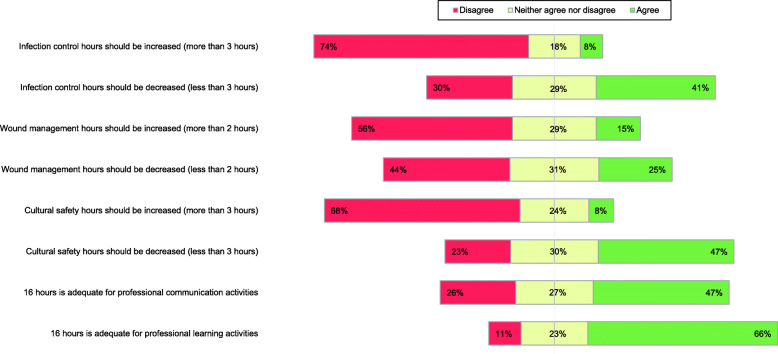
Perceptions of the time requirements for compulsory CPD activities

Figure 3 details the respondent’s perceptions of the time requirements for compulsory CPD activities. Overall, respondents disagreed with increasing CPD hours for infection control (*n* = 96, 74 %) and cultural safety (*n* = 88, 68 %). However, when asked about wound management, just over half of respondents (*n* = 73, 56 %) disagreed that CPD hours should be increased. 47 % of respondents (*n* = 63) agreed that the 16-h allocation towards professional communication activities was adequate. Regarding professional learning activities, two-thirds of respondents (*n* = 88, 66 %) agreed that 16 h is adequate.

#### Barriers to CPD participation

**Fig. 4:**
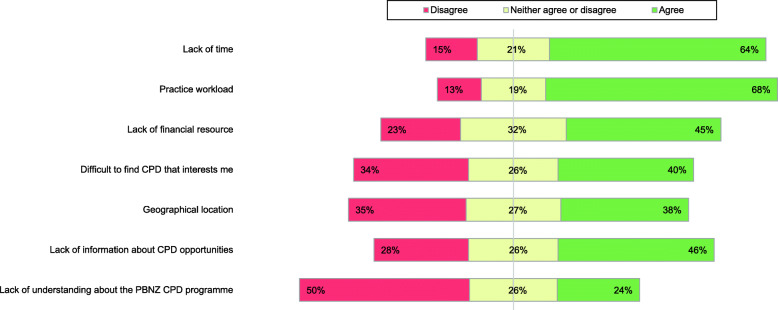
Barriers to CPD participation

Figure 4 details the barriers to CPD participation. Lack of time (*n* = 84, 64 %) and clinical practice workload (*n =* 90, 68 %) were the strongest barriers. Lack of financial resources (*n =* 60, 45 %) and lack of information about CPD opportunities (*n* = 61, 46 %) were cited as additional barriers to participation. In addition, 40 % (*n* = 53) agreed that it is difficult to find CPD of interest to them and 38 % (*n* = 50) reported that geographic location is a barrier. Half of respondents (*n* = 66, 50 %) disagreed that lack of understanding of the current CPD programme is a barrier to CPD participation.

### Qualitative analysis

There were 40 useable responses to question 16, “Please describe why you found it difficult to complete the [CPD] requirements”. Three categories encompassing six subcategories were identified from the qualitative data analysis: understanding CPD; access to CPD; and time to complete CPD. Illustrative quotes from participant responses have been selected to represent each sub-category.

#### Understanding CPD

Responses describing difficulty understanding CPD were grouped into two subcategories: understanding the new CPD programme; and difficulties navigating the online platform. In terms of the CPD programme, there was apparent difficulty interpreting the CPD categories. Respondents described a “lack of understanding of what was required…” and “being used to the old way of doing things”. One participant admitted to manipulating CPD activities to fit the framework:

*“I hadn’t recorded all the CPD I had done, and I found some of the categories limiting, so in the end you manipulate your CPD to fit, which I doubt is the intention, but what you have to do to make it all fit.” (part-time podiatrist with greater than 21 years experience)*.

Respondents also indicated that “finding the correct number of hours in some of the categories was difficult as some of the categories are uninteresting.” This was particularly challenging for the compulsory CPD categories such as wound care, with one respondent stating:

*“Wound care management is not my area of interest or what I choose to specialise in. I found the compulsory areas of CPD in this regard stressful to gather enough points together.” (part-time podiatrist with greater than 21 years experience)*.

Difficulties navigating the online system were also apparent and attributed to a “lack of technology skills”, by many respondents. One participant summed this up stating:

“[online] CPD platforms were difficult to understand and use.” *(part-time podiatrist with 0–2 years experience)*.

#### Access to CPD

Responses concerning access to CPD were divided into two subcategories relating to: difficulty accessing CPD courses; and difficulty accessing colleagues for support and networking. Regarding access to CPD courses, respondents described their rural location as a barrier, believing that “most things [courses] are run in the main centres” and there are “not enough local learning opportunities”. Working as a sole practitioner was also a significant barrier with many podiatrists “working alone [and] isolated.”

*“As a sole practitioner [I] feel unsupported with no professional body to assist.” (full-time podiatrist with 3–5 years experience)*.

*“Some requirements are hard to achieve. Need to have students or be part of meetings. Hard when you’re the only pod [podiatrist] in the village.” (full-time podiatrist with 3–5 years experience)*.

Respondents also identified the cost associated with courses, including the cost of not working in order to attend courses, as barriers. “Cost and time away from clinic can make attending courses difficult due to travel and extra expenses”. Frustration was also directed at cost imposed by those who run CPD courses:

*“They are all so expensive. With today’s living costs and very low podiatry salaries, the over-charged courses are a rort and show bad faith of the Podiatry Board.” (part-time podiatrist with 3–5 years experience)*.

Access to courses both face-to-face and online was also an issue with one participant expressing the belief that the University [AUT] delivering the podiatry undergraduate degree should also provide CPD activities:

*“Lack of CPD events and virtually nothing offered by the undergraduate education provider. Meaning a grave lack of connection between the School of Podiatry and the profession.” (part-time podiatrist with greater than 21 years experience)*.

*“I think we should get more online courses to manage our CPD.” (part-time podiatrist with 6–10 years experience)*.

A further barrier to accessing CPD was attributed to finding courses that are of interest or relevance to fit into the categories:

*“Some areas of CPD are difficult to find good, relevant content. Some of this is because my computer skills are not great and that is frustrating at times.” (part-time podiatrist with 16–20 years experience)*.

*“Sometimes finding things that are applicable to my practice can make me less motivated too.” (full-time podiatrist with greater than 21 years experience)*.

In relation to the second subcategory ‘difficulty accessing colleagues for support and networking’, this was particularly evident in rural NZ where there is “limited access to regular peer groups and meetings.”

*“Quite honestly found it difficult to network and meet with colleagues in rural, regional practice.” (full-time podiatrist with 3–5 years experience)*.

*“I didn’t understand how much I had to do in the company of other podiatrists, CPD is not something you can do alone, you have to belong to a team of people you can trust. Working in a small community it can be very uncomfortable to work with ‘the opposition’ in town.” (full-time podiatrist with greater than 21 years experience)*.

#### Time to complete CPD

Responses concerning the time to complete CPD were divided into two subcategories: too busy to complete CPD; and leaving it too late. Respondents described the pressures of running a business combined with family commitments made it difficult to make time for CPD. Those who worked as sole practitioners also described time difficulties associated with no-one able to cover their clinical patient load when they attended CPD activities.

*“Finding time to attend and record all these and juggling time for patients and family matters has been just too difficult and challenging, especially with lack of support.” (part-time podiatrist with 11–15 years experience)*.

*“Time is also an issue, as I am sole podiatrist, I have no staff to delegate some jobs to, so keeping up with managing my practice and clinic doesn’t leave me with a lot of time.” (full-time podiatrist with greater than 21 years experience)*.

Several respondents admitted to leaving it too late to complete the CPD activities within the two-year cycle. “Not being up to date with the latest changes to the new CPD framework” contributed to time pressures as well as the time associated with “either sourcing activities or travel”.

## Discussion

Very little is known about the perceptions of NZ podiatrists towards mandatory CPD. This study presents the first known data collected about the perceptions towards CPD and the satisfaction with the CPD programme.

The survey data indicated NZ podiatrists agreed that it is important to engage in CPD and that CPD helps maintain their competence. Although respondents linked CPD to professional competence, there is limited evidence to support the supposition that undertaking regular CPD leads to the advancement of professional competence [5, 6]. This criticism stems from the belief that CPD based on attainment of a certain number of hours implies practitioners must only provide evidence of their attendance or participation in a CPD activity [6]. In the context of the NZ CPD programme, evidence is not required to support the relevance of the CPD activity to the needs of the podiatrist, their level of active participation in the activity, or whether the knowledge gained through undertaking the activity is applied to practice. Therefore, the effectiveness and usefulness of the CPD activity is unknown. Consequently, CPD programmes may be a weak proxy for competency and as postulated by Lysaught et al. “the primary value of CPD only be as a reminder to members that maintaining competency is a requirement of practice” [6].

**Fig. 5:**
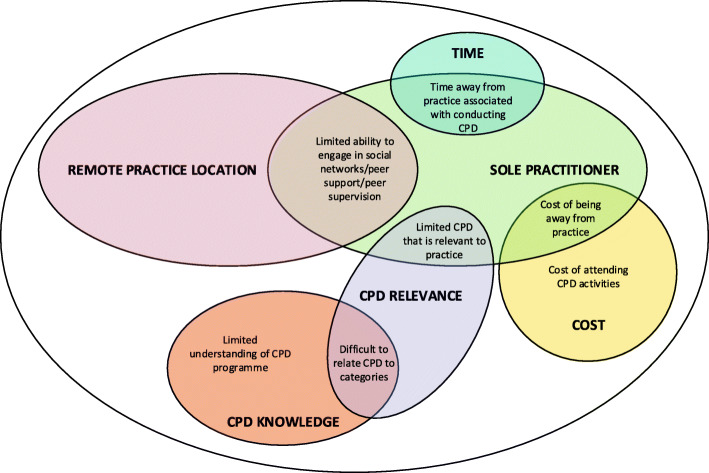
Elements affecting practitioner engagement in CPD

In agreement with findings from CPD studies in radiographers [7], nurses [8], and pharmacists [9], a lack of time, practice workload, and lack of financial resources are primary factors that influence the capacity to engage in CPD. However, the capacity for practitioners to engage in CPD is also affected by numerous interrelated elements (Fig. 5). Two major elements being geographical practice location and employment context, such as sole or group private practice. NZ podiatry workforce data indicates approximately 35 % of podiatrists work outside the three main metro areas (Auckland, Wellington and Christchurch), with 80 % of the profession in private practice and 60 % either self-employed or a business owner [9]. In the current study, qualitative data highlighted those practitioners in remote areas reported having limited access to social/peer support networks, had limited opportunities to meet face to face, and were faced with additional issues of travel time and distance to attend organised CPD meetings. Locality issues are further intensified by the employment context. Practitioners working in a sole practitioner environment may find CPD engagement difficult due to their limited ability to participate in social interaction during daily practice. Subsequently limiting their ability to engage in essential activities such as observational learning, imitation, peer discussion, and ‘reflective conversation [10]. Conversely, podiatrists working in large organisations (universities and District Health Boards), representing two and eight % of the NZ profession respectively [9], may not be faced with these issues due to easier access to a variety of workplace-based CPD opportunities, protected time to undertake CPD, and CPD funding built into work contracts.

The PBNZ CPD programme has mandated compulsory elements of infection control, wound management, and cultural safety which were also components of the original CPD framework (2004 to 2017). In the current study, data indicated dissatisfaction with hours attributed to the compulsory activities. Hours based CPD frameworks have been criticised for their counting of hours of learning, not performance; for only measuring participation; for their focus on quantity not quality; paying inadequate attention to helping individuals improve their own practice; and their lack of promotion of collaboration [11, 12]. This criticism may be best embodied in the participant responses to the compulsory cultural safety requirement of the PBNZ CPD programme. In the context of NZ healthcare, there is growing recognition of the importance of cultural safety at both individual health practitioner and organisational levels to achieve equitable health outcomes [13]. However, respondents indicated the hours towards cultural safety should be decreased (Fig. 4). On the surface this may be an alarming finding, however, it may not reflect NZ podiatrists’ under-valuing cultural safety. Rather, it may indicate that such a complex concept cannot be simply and meaningfully undertaken as a CPD activity within an hours-based framework. This may suggest that the approach to cultural safety CPD should not be compressed into a time-based approach but rather embedded in a deeper level of CPD that promotes an understanding and relevance to practice, moving towards behavioural change.

One mechanism by which the identified barriers to CPD may be reduced, and the perceived relevance of CPD increased, is through shifting the focus of learning back into the practitioner’s working environment. This contrasts with viewing CPD as something that needs to be conducted away from the workplace. This shift may better align with the individual practitioner’s work within their ‘Scope of Practice’. Although the Scope of Practice for NZ podiatrists is tightly defined, there must be recognition that an individual’s scope of practice also expands over the path of their career. Factors that influence scope expansion include, employment progression/promotion, specialisation within their area of practice, and the wider range of tasks assumed in their practice setting, which largely evolve in response to addressing the changing needs of their patients [14, 15]. Accordingly, an evolving CPD programme needs to reflect the changing breadth and complexity of an individual’s professional practice and career development, to enable freedom to undertake CPD viewed as relevant to the individual’s learning needs.

This study is limited by the depth by which the CPD programme was evaluated, only evaluating participant satisfaction. Consequently, the impacts (multiple and varied short, medium, and long-term outcomes) of the CPD programme on the personal and professional practices of NZ podiatrists remains unexplored and are largely unknown. Interpretation of the data is also limited by the response rate with only one-third of NZ registered practitioners with a practising certificate responding to the survey. The timing of the survey must also be given consideration; it was distributed following COVID-related lockdowns and this may have influenced the level of engagement with this topic. Despite these limitations, this is the first data set related to CPD and NZ podiatry. Future research is required to understand how CPD changes attitudes, knowledge and skills, and behaviours related to learning and practice. Issues that directly affect the process of learning and the relationship to CPD participation - such as learning barriers, language, physical health, learning difficulties, and social and personal circumstances - also require consideration. Future research should also consider the effects of increasing virtual learning opportunities such as webinars and live discussion groups that have evolved due to COVID. Particularly if these virtual learning opportunities have enabled the development of peer support and mentorship networks for the sole practitioner in private practice.

## Conclusions

NZ podiatrists value CPD and are satisfied with most aspects of the mandatory CPD programme apart from the hours attributed to compulsory activities. The current approach to cultural safety CPD requires revision, with a move away from a time-based approach to a system that promotes an understanding and relevance to practice. Lack of time, practice workload, financial barriers, geographical location, and employment context were factors that influenced a practitioner’s ability to engage in CPD. Facilitation of CPD activities that are flexible to ensure relevance to the practitioner’s specific work within their scope of practice, and that can occur in the workplace environment, may address barriers and increase engagement with to CPD activities.

## Supplementary Information

**Additional file 1.** Overview of the PBNZ CPD recertification framework for NZ podiatrists.

**Additional file 2.** Checklist for Reporting Results of Internet E-Surveys (CHERRIES).

## Data Availability

Request for further details of the data set and queries relating to data sharing arrangements may be submitted to Matthew Carroll (matthew.carroll@aut.ac.nz). The survey does not obtain consent for participant data to be shared, although the present data are anonymised with all personal identifiers removed.

## Abbreviations

CHERRIES: Checklist for Reporting Results of Internet E-Surveys
CPD: Continuing professional development
NZ: Aotearoa New Zealand
PBNZ: Podiatrists Board of New Zealand
APC: Annual practising certificate
UK: United Kingdom
