# Supplementary material for: Perceptions and satisfaction of a mandatory continuing professional development programme amongst Aotearoa New Zealand podiatrists
Source: J Foot Ankle Res. 2021 Sep 8;14:54. doi: 10.1186/s13047-021-00492-6 (PMC8424971; doi:10.1186/s13047-021-00492-6)

Perceptions and satisfaction of a mandatory continuing professional development programme amongst Aotearoa New Zealand podiatrists

Additional File 1: Overview of the PBNZ CPD recertification framework for NZ podiatrists

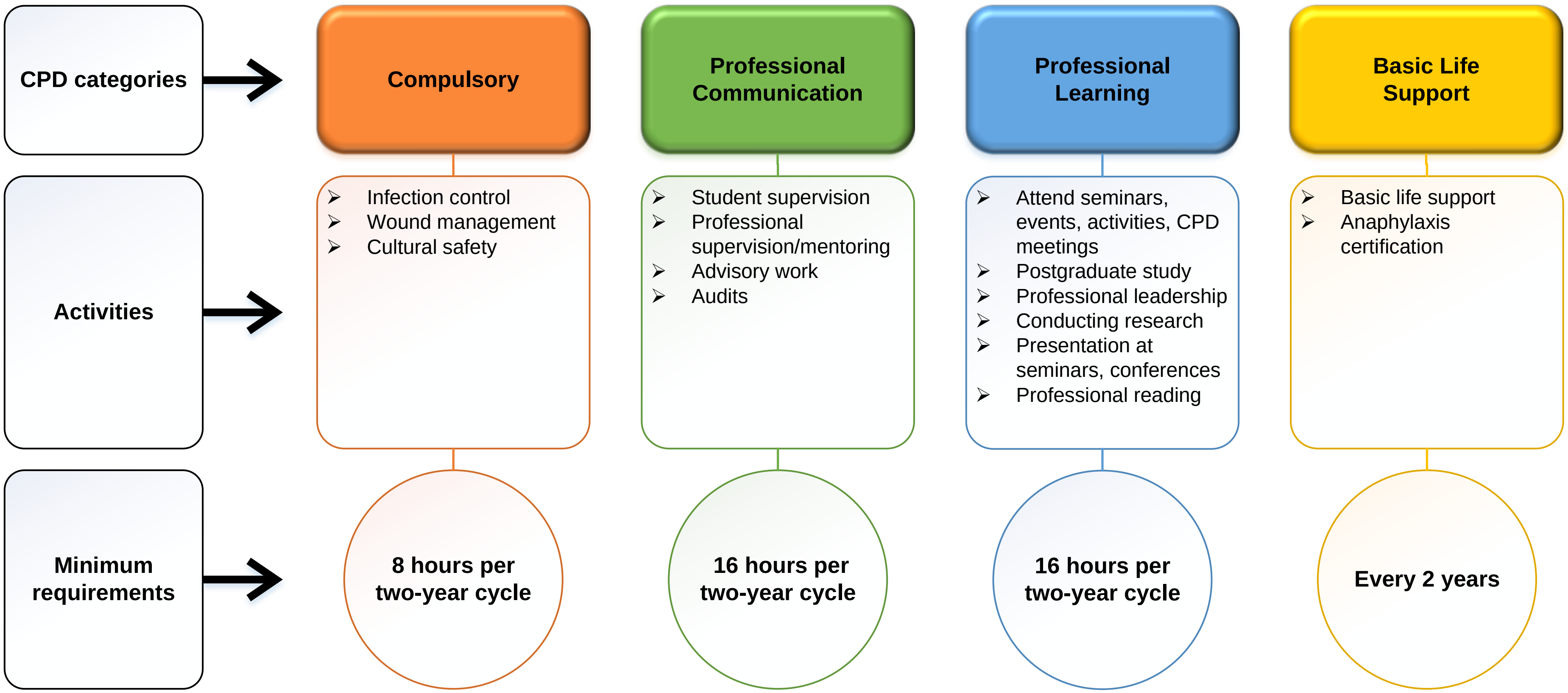

Supplement: Supplementary file 1 — Additional file 1. Overview of the PBNZ CPD recertification framework for NZ podiatrists. [file 13047_2021_492_MOESM1_ESM.pdf]
